# Clinical and Radiological Outcomes of Organising Pneumonia Secondary to COVID-19: A 2-Year Longitudinal Cohort Study

**DOI:** 10.3390/jcm15186999

**Published:** 2026-09-10

**Authors:** Oswaldo Antonio Caguana-Vélez, Diana Badenes-Bonet, Xavier Duran, Flavio Zuccarino, Didac Ramal, Santiago Carbullanca, Judit Villar-García, Diego A. Rodríguez-Chiaradía, Eva Balcells

**Affiliations:** 1 Respiratory Medicine Department, Hospital del Mar, 08003 Barcelona, Spain; dbadenes@hmar.cat (D.B.-B.); darodriguez@hmar.cat (D.A.R.-C.); ebalcells@hmar.cat (E.B.); 2 Department of Medicine and Life Sciences (MELIS), Universitat Pompeu Fabra (UPF), 08003 Barcelona, Spain; fzuccarino@hmar.cat (F.Z.); jvillar@hmar.cat (J.V.-G.); 3 Hospital del Mar Medical Research Institute, 08003 Barcelona, Spain; 4 Medical Statistics Unit, Hospital del Mar Medical Research Institute, 08003 Barcelona, Spain; xduran@researchmar.net; 5 Department of Radiology, Hospital del Mar, 08003 Barcelona, Spain; dramal@hmar.cat (D.R.); scarbullanca@hmar.cat (S.C.); 6 Department of Radiology, Hospital Sant Joan de Déu, Esplugues de Llobregat, 08950 Barcelona, Spain; 7 Infectious Diseases Department, Hospital del Mar, 08003 Barcelona, Spain; 8 Centro de Investigación en Red de Enfermedades Respiratorias, (CIBERES), Instituto de Salud Carlos III (ISCIII), 28029 Madrid, Spain

**Keywords:** COVID-19, organizing pneumonia, high-resolution computed tomography, fibrotic-like lesions, long-term follow-up

## Abstract

**Background**: Organising pneumonia secondary to COVID-19 (OP-COVID-19) is a recognised inflammatory complication of SARS-CoV-2 infection. We assessed the 2-year clinical, radiological, and functional evolution of patients hospitalised with OP-COVID-19 and identified factors independently associated with long-term fibrotic-like lesions. **Methods**: This ambispective longitudinal study included adults consecutively hospitalised with OP-COVID-19 from March 2020 to February 2021. Diagnosis was based on clinical assessment and high-resolution computed tomography (HRCT). Patients underwent standardised follow-up at 3, 6, 12, and 24 months after hospital discharge, including clinical assessment, chest HRCT, pulmonary function tests, and the six-minute walk test. Longitudinal radiological changes were assessed using paired comparisons, and exploratory multivariable logistic regression identified factors independently associated with long-term fibrotic-like lesions. **Results**: OP-COVID-19 was diagnosed in 271 patients (4.9%), and 228 were included in the follow-up cohort. Inflammatory HRCT abnormalities resolved progressively, with paired comparisons showing a significant reduction in consolidations, peribronchovascular opacities, crazy-paving pattern, reversed halo sign, and perilobular opacities at 3–6 months compared with the initial HRCT (*p* < 0.05 for all). Among the 221 patients with an ascertainable final radiological outcome, persistent pulmonary abnormalities were observed in 14.9%, including fibrotic-like lesions in 10.4%. No statistically detectable late radiological progression was observed among patients undergoing extended imaging follow-up. The need for respiratory support during the acute phase (odds ratio [OR] 5.79, 95% confidence interval [CI] 1.81–18.52), bronchial dilatation (OR 3.50, 95% CI 1.10–11.11), and architectural distortion and/or volume loss on initial HRCT (OR 4.72, 95% CI 1.80–12.35) were independently associated with long-term fibrotic-like abnormalities. Pulmonary function was largely preserved, with mildly reduced diffusing capacity for carbon monoxide (DLCO) in the assessed subsets. **Conclusions**: OP-COVID-19 has a favourable long-term prognosis, with progressive resolution of inflammatory abnormalities in most patients. Fibrotic-like lesions occur in a minority of patients and are associated with greater initial disease severity and early structural abnormalities on HRCT. These findings support risk-stratified follow-up for patients with OP-COVID-19.

## 1. Introduction

The COVID-19 pandemic, caused by severe acute respiratory syndrome coronavirus 2 (SARS-CoV-2), has imposed a substantial burden of respiratory illness worldwide [1]. Among its pulmonary complications, organising pneumonia (OP) is a well-recognised entity. It is an inflammatory and fibroproliferative process characterised by intraalveolar fibroproliferation that is reversible with (and occasionally without) immunosuppressive or anti-inflammatory therapy [2]. OP can develop under various clinical conditions, including infections, drug reactions, connective tissue disorders, and malignancies [2,3,4,5].

Organising pneumonia secondary to COVID-19 (OP-COVID-19) has been described as a post-acute manifestation following the initial phase of the disease, primarily with the first circulating variants [6]. Clinically, it is characterised by persistent cough and dyspnoea, often requiring supplemental oxygen or respiratory support [5]. High-resolution computed tomography (HRCT) of the chest is crucial for identifying the OP pattern. Characteristic radiological findings of this pattern include parenchymal consolidations and ground-glass opacities (GGO), which are typically bilateral and multifocal, predominantly affecting the peripheral regions of the lower lobes and often associated with subpleural parenchymal bands [2,3,4]. Although the diagnosis of OP generally relies on the integration of clinical, radiological, and sometimes histopathological findings, in the context of prior SARS-CoV-2 infection it is commonly established based on a compatible clinical presentation and HRCT features, given the limited feasibility of lung biopsy in this population [2,3,4].

To date, most studies on OP-COVID-19 have been retrospective or limited to short-term follow-up [7,8,9,10], but prospective data beyond the first year are lacking, and previous studies have rarely integrated clinical, radiological, and functional trajectories or explored predictors of fibrotic evolution within a prospective framework.

The primary objective of this study was to assess the long-term clinical, radiological, and functional evolution of patients hospitalised with OP-COVID-19 over a two-year follow-up period. As a secondary objective, we aimed to identify which of these characteristics were associated with long-term fibrotic-like lesions.

## 2. Materials and Methods

### 2.1. Study Design and Population

This was a single-centre ambispective longitudinal cohort study conducted at a tertiary hospital. Adults aged over 18 years hospitalised between March 2020 and February 2021 with a diagnosis of SARS-CoV-2 infection who developed respiratory complications consistent with OP were included. SARS-CoV-2 infection was confirmed by polymerase chain reaction testing of nasopharyngeal swabs. All included patients were experiencing their first documented SARS-CoV-2 infection, and no previous SARS-CoV-2 infections were identified. During the study period, the predominant circulating variant was lineage B.1.1.7 (88%), followed by B.1.177/A222V (7.5%). Given the inclusion period, most patients were enrolled before widespread SARS-CoV-2 vaccination coverage. Patients with pre-existing interstitial lung disease (ILD) or those who declined to participate in the study were excluded.

Patients with persistent or worsening respiratory symptoms, such as cough, dyspnoea, or continued need for oxygen therapy or other respiratory support, at 14 days after symptom onset, were evaluated by a pulmonologist for suspected OP-COVID-19 according to the institutional protocol. The diagnosis of OP-COVID-19 was based on clinical assessment, laboratory findings, and HRCT features. Patients received intravenous corticosteroid therapy with methylprednisolone (1 mg/kg/day) or dexamethasone (6, 9, or 12 mg/day), according to disease severity. Intravenous corticosteroids were maintained until clinical improvement and a reduction in oxygen requirements were observed. Treatment was subsequently switched to oral prednisone at 0.5 mg/kg/day for 2 weeks, followed by dose reductions of 10 mg/day every 2 weeks until discontinuation. The tapering period was extended when persistent symptoms or radiological abnormalities were observed.

**Figure 1:**
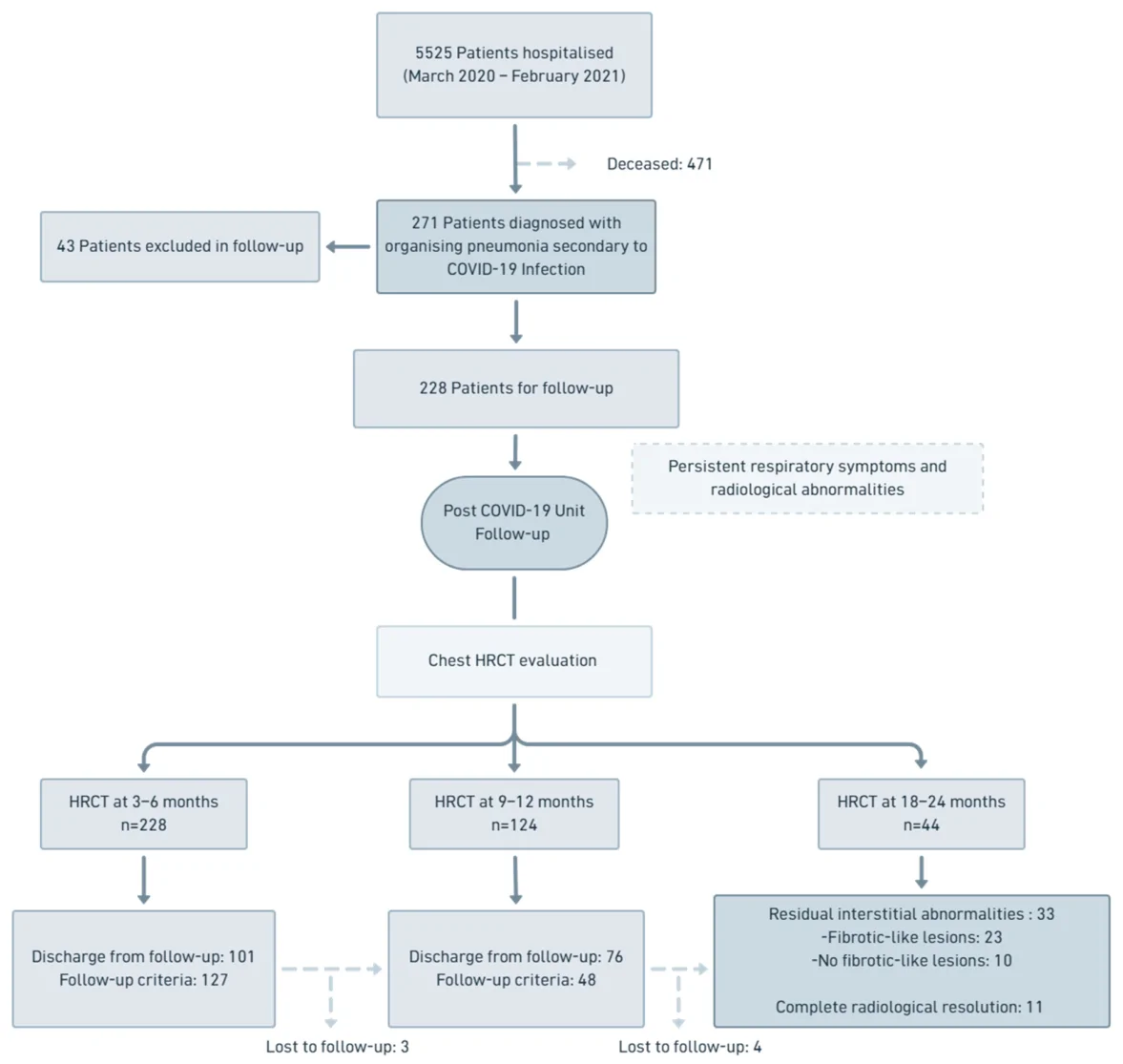
Flowchart of patient selection and longitudinal follow-up of patients with organising pneumonia secondary to COVID-19. No fibrotic-like lesions were defined by the presence of ground-glass opacities, reticular pattern, and/or parenchymal bands, whereas fibrotic-like lesions included honeycombing, bronchial dilatation/traction bronchiectasis, volume loss, and/or architectural distortion. Abbreviations: HRCT: high-resolution computed tomography.

A screening follow-up telephone visit was conducted 1–2 weeks after hospital discharge to assess clinical evolution through a structured symptom checklist. According to disease severity during hospitalisation and clinical status after discharge, patients were scheduled for an early in-person evaluation within the first 1–3 months after discharge, including blood tests and chest X-ray assessment. Subsequent follow-up visits were performed at 3, 6, 9, 12, and 24 months in a specialised multidisciplinary post-COVID unit. These visits included symptom assessment, chest HRCT, pulmonary function tests (PFTs), and the six-minute walk test (6MWT), according to a standardised follow-up protocol (shown in Figure 1). Dyspnoea severity was subsequently assessed during follow-up visits using the modified Medical Research Council (mMRC) dyspnoea scale.

### 2.2. Clinical Data Collection and Laboratory Tests

Data on age, sex, smoking status, and comorbidities (obesity, dyslipidaemia, arterial hypertension, diabetes mellitus, chronic obstructive pulmonary disease (COPD), asthma, bronchiectasis, coronary artery disease, atrial fibrillation, chronic kidney disease, cancer, and immunosuppression) were obtained from electronic medical records at admission. Autoimmune disease and chronic baseline treatments were not systematically recorded. We also recorded the length of stay, type of respiratory support (high-flow oxygen therapy, non-invasive or invasive ventilation, continuous positive airway pressure), intensive care unit (ICU) admission, and corticosteroid treatment (type, dose, and duration). Blood samples were analysed for inflammatory markers at the time of OP-COVID-19 diagnosis, including C-reactive protein (CRP), interleukin-6 (IL-6), leukocyte count, lactate dehydrogenase (LDH), ferritin, N-terminal pro-brain natriuretic peptide (NT-proBNP), and D-dimer. At discharge, baseline oxygen saturation (SpO_2_) and need for home oxygen therapy were evaluated.

### 2.3. Radiological Evaluation

HRCT of the chest was used to assess pulmonary parenchymal involvement. Findings were independently evaluated by two thoracic radiologists with expertise in ILD. In patients with clinical suspicion of pulmonary embolism, computed tomography (CT) pulmonary angiography was performed, and lung images were used to evaluate lung parenchyma. HRCT images were reconstructed with a slice thickness of 1 to 3 mm and reviewed in a lung window: window width of 1000 to 2000 Hounsfield units; window level of −700 to −500 Hounsfield units.

Analysis focused on radiological features consistent with an OP pattern, including patchy or confluent parenchymal consolidations, GGO, and bilateral, peripheral, or peribronchial distribution, especially in the middle and lower lobes. Characteristic OP signs, such as the reversed halo sign and perilobular consolidations (arcade-like), were specifically assessed. Fibrotic-like lesions, including honeycombing, traction bronchiectasis/bronchiolectasis, architectural distortion, and volume loss, were systematically evaluated [11,12]. Findings were reviewed by clinical-radiological consensus in the multidisciplinary post-COVID-19 unit.

HRCT evaluations were performed at OP-COVID-19 diagnosis and subsequently at 3–6, 9–12, and 18–24 months to document persistent pulmonary parenchymal lesions. Follow-up schedules were adjusted according to radiological findings and the clinical evolution of each patient. In addition, exploratory paired comparisons between 9–12 and 18–24 months were conducted in the subset of patients with imaging available at both time points.

### 2.4. Functional Follow-Up

At 3–6 and 18–24 months after hospitalisation for OP-COVID-19, PFTs and the 6MWT were performed in accordance with the American Thoracic Society (ATS) and the European Respiratory Society guidelines [13,14]. Pulmonary function included forced spirometry and diffusion capacity for carbon monoxide (DLCO). Tests were indicated based on clinical judgement, considering the persistence of respiratory symptoms and/or parenchymal abnormalities identified on imaging.

### 2.5. Statistical Analysis

Categorical variables were expressed as frequencies and percentages, whereas continuous variables were presented as mean (standard deviation) or median (interquartile range, IQR), depending on their distribution. Normality was assessed using the Shapiro–Wilk test. Comparisons between groups were performed using Pearson’s chi-squared test for categorical variables and unpaired Student’s t-test for normally distributed continuous variables, or the Mann–Whitney U test when normality could not be assumed.

The evolution of main radiological findings over time was analysed in longitudinal paired data by comparing paired proportions with McNemar’s exact test. As no adjustment for multiple comparisons was applied, these longitudinal analyses were considered exploratory.

To identify factors independently associated with the presence of fibrotic-like lesions, a multivariable logistic regression analysis was conducted. Variables entered in the exploratory multivariable model were selected based on clinical relevance and statistical significance (*p* < 0.10) in the univariable analyses. The analysis included all 221 patients with an ascertainable final radiological outcome, including 23 patients with fibrotic-like lesions and 198 without fibrotic-like lesions. No missing data were present for the variables included in the final model; therefore, no imputation or additional exclusion due to missing data was required. Multicollinearity among predictors was assessed using tolerance and variance inflation factor (VIF) values. Odds ratios (ORs) with 95% confidence intervals (CIs) were calculated. Statistical significance was set at a two-sided *p*-value ≤ 0.05. All analyses were conducted using IBM SPSS Statistics, version 28.0 (IBM Corp., Armonk, NY, USA).

#### Use of Generative Artificial Intelligence

During manuscript preparation and revision, ChatGPT (GPT-5.5 and GPT-5.6; OpenAI, San Francisco, CA, USA) was used for language editing and to improve clarity and readability. It was not used for data collection or statistical analysis. All AI-assisted content was reviewed and edited by the authors, who take full responsibility for the final manuscript.

### 2.6. Ethical Approval

The study was conducted in accordance with the principles of the Declaration of Helsinki and was approved by the local Ethics Committee (No. 2020/9455). The post-COVID-19 clinic was established as part of routine care following hospital discharge. Data collected before ethics approval were retrospectively retrieved from medical records, whereas subsequent follow-up data were prospectively collected in accordance with the approved protocol. Patient confidentiality was maintained, and the Ethics Committee waived the requirement for written informed consent because anonymised routine care data were used during the exceptional circumstances of the COVID-19 pandemic.

## 3. Results

### 3.1. Patient Characteristics

Of the 5525 patients hospitalised with COVID-19 during the study period, 271 (4.9%) were diagnosed with OP-COVID-19. During the post-hospitalisation follow-up, 7 patients died, and 36 were lost to follow-up. In total, 228 patients were included in the post-discharge follow-up analysis. Seven additional patients were lost during longitudinal follow-up, resulting in 221 patients completing follow-up (Figure 1). The clinical characteristics of patients with OP-COVID-19 at admission are summarised in Table 1. The mean age was 64.7 years, 67.5% were male, and 68.3% had never smoked. The most frequent comorbidities were hypertension, dyslipidaemia, and diabetes mellitus. A minority of patients had pre-existing respiratory diseases, including COPD (9.6%), asthma (4.8%), and bronchiectasis (1.9%). Among the 15 patients with a history of cancer, none was receiving active cancer-specific systemic treatment during the study period.

The median (IQR) length of hospital stay was 24 (16–33) days, with 31% requiring ICU admission. At OP-COVID-19 diagnosis, 46.1% of the patients needed respiratory support. Laboratory analyses during hospitalisation showed elevated inflammatory markers, including ferritin, fibrinogen, CRP, D-dimer, IL-6, NT-proBNP and LDH (Table 1).

### 3.2. Chest HRCT Findings and Temporal Evolution

The HRCT findings at OP-COVID-19 diagnosis and during follow-up are described in Table 2. In the initial HRCT performed at OP-COVID-19 diagnosis, the most frequent findings were GGO (99.3%), consolidations (98.9%), peribronchovascular opacities (84.5%), parenchymal bands (77.9%), reticular pattern (63.5%), and crazy-paving pattern (54.2%). Less common findings included reversed halo sign (30.6%), architectural distortion or volume loss (30.3%), bronchial dilatation (11.1%), mosaic pattern (10.0%), and perilobular opacities (6.0%).

Paired longitudinal analyses were performed separately between the initial HRCT and each available follow-up time point (Supplementary Table S1). At 3–6 months (*n* = 228 paired examinations), McNemar’s test showed significant reductions in several predominantly inflammatory findings, including consolidations, peribronchovascular opacities, parenchymal bands, reticular pattern, crazy-paving pattern, reversed halo sign, and perilobular opacities (*p* < 0.05 for all). Of the 228 patients who entered post-discharge follow-up, seven were subsequently lost to follow-up, leaving 221 patients with an ascertainable final radiological outcome. Among these, 44 patients with persistent radiological abnormalities underwent extended imaging follow-up at 18–24 months. Of these, 33 patients (75%) continued to show lung abnormalities at 18–24 months, including 23 (69.7%) with fibrotic-like lesions, defined as traction bronchiectasis or bronchiolectasis and/or architectural distortion with volume loss. The remaining 10 patients (30.3%) showed only ground-glass opacities, reticulation, or parenchymal bands. Overall, at 18–24 months, among patients with an ascertainable final radiological outcome, persistent lung abnormalities were observed in 33/221 (14.9%), including fibrotic-like lesions in 23/221 (10.4%). Among the 44 patients undergoing extended imaging follow-up, 23 (52.3%) showed fibrotic-like lesions at 18–24 months.

When comparing paired HRCT findings at 9–12 and 18–24 months of follow-up (*n* = 44), McNemar’s test showed no statistically significant changes in most radiological findings. Specifically, subpleural parenchymal bands showed a significant reduction from 9–12 to 18–24 months (*p* = 0.002). No statistically significant changes were observed for GGO and traction bronchiectasis (*p* = 0.065 and *p* = 0.063, respectively). No significant changes were observed between 9–12 and 18–24 months in peribronchovascular opacities, reticular pattern, crazy-paving pattern, architectural distortion or volume loss, or mosaic attenuation.

**Figure 2:**
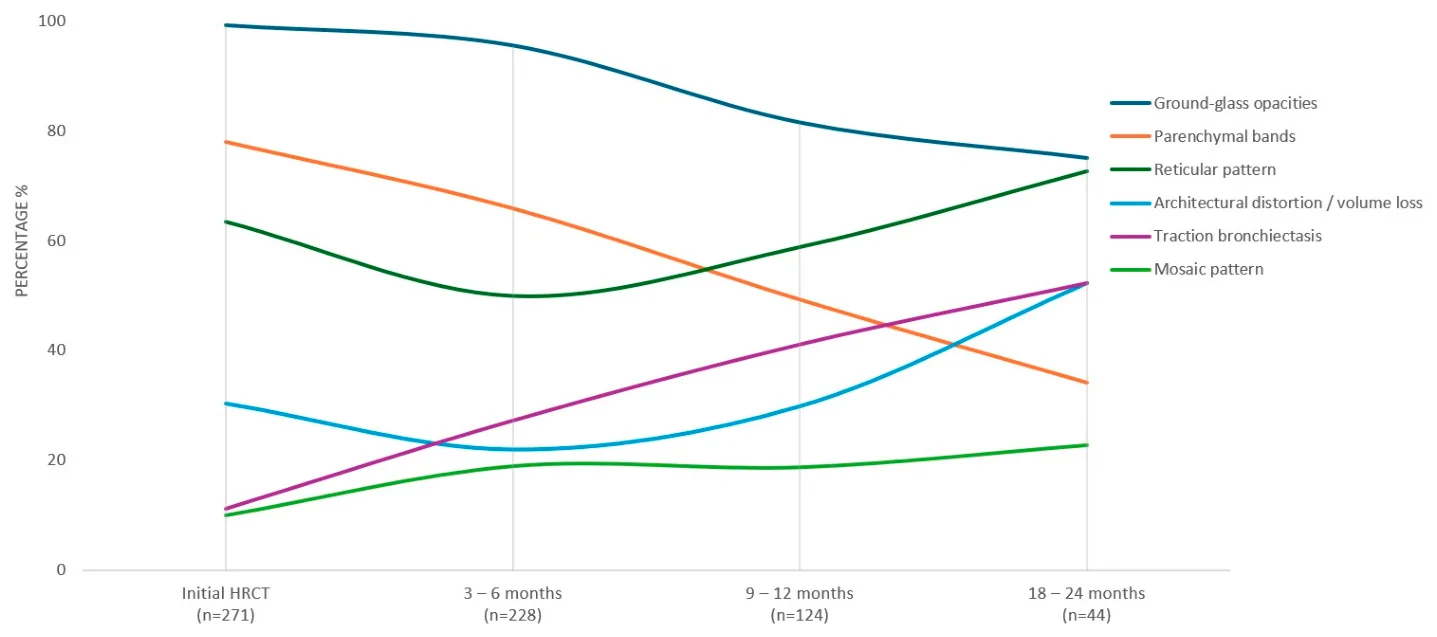
Temporal evolution of major radiological abnormalities during follow-up in patients with OP-COVID-19. The figure shows the percentage of patients with selected radiological findings on HRCT at baseline and during follow-up. Follow-up HRCT was selectively performed in patients with persistent radiological abnormalities; therefore, the number of patients assessed and the corresponding denominators differed across time points. Percentages should not be interpreted as longitudinal changes within the same patient population.

Among the 221 patients with an ascertainable final radiological outcome, 23 had fibrotic-like lesions, and 198 did not. The latter group included patients with residual non-fibrotic abnormalities at 18–24 months and those whose HRCT findings had normalised during follow-up. Seven patients lost to follow-up were excluded from these analyses because their final radiological outcome could not be ascertained (Table 3 and Table 4). Patients with fibrotic-like lesions were older (70.1 vs. 64.4 years, *p* = 0.039), had longer hospital stays (37 [30–49] vs. 22 [16–31] days, *p* = 0.001), and a higher proportion required respiratory support (82.6% vs. 43.9%, *p* < 0.001) or ICU admission (73.9% vs. 29.8%, *p* < 0.001). In the HRCT performed at OP-COVID-19 diagnosis, this group more frequently exhibited a reticular pattern (87% vs. 60%, *p* = 0.012), a crazy-paving pattern (78.3% vs. 51.0%, *p* = 0.013), involvement of all lung fields (100% vs. 74.6%, *p* = 0.003), bronchial dilatation (30.4% vs. 7.6%, *p* = 0.003), and architectural distortion or volume loss (60.9% vs. 20.7%, *p* < 0.001). The temporal evolution of the main radiological abnormalities during follow-up is shown in Figure 2.

In the exploratory multivariable logistic regression analysis, three factors were independently associated with fibrotic-like lesions at 18–24 months: the need for respiratory support during the acute phase (OR = 5.79; 95% CI: 1.81–18.52; *p* = 0.003), the presence of bronchial dilatation in the HRCT performed at OP-COVID-19 diagnosis (OR = 3.50; 95% CI: 1.10–11.11; *p* = 0.033), and the presence of architectural distortion or volume loss (OR = 4.72; 95% CI: 1.80–12.35; *p* = 0.002) observed in the initial HRCT performed at OP-COVID-19 diagnosis.

Multicollinearity diagnostics showed high tolerance values (0.942–0.988) and variance inflation factor values close to 1 (1.012–1.062), indicating the absence of relevant collinearity among predictors included in the final model (Table 5).

### 3.3. Clinical and Functional Follow-Up

After hospital discharge, 7.4% of patients required home oxygen therapy, which was discontinued within the first month in all cases. At the 3–6-month visit, 57.4% of patients reported an mMRC dyspnoea score of 0, 38.6% a score of 1, and 4.0% a score of ≥2. At the final follow-up, among the 23 patients with fibrotic-like lesions, 82.6% had an mMRC dyspnoea score of ≥1 and 4.3% had a score of ≥2. PFTs were performed at 3–6 months in 88 patients, all of whom had complete data for the reported pulmonary function parameters, with mean predicted forced expiratory volume in one second (FEV_1_), forced vital capacity (FVC), FEV_1_/FVC, and DLCO values of 85.0%, 86.3%, 77.4%, and 76.0%, respectively. At 18–24 months, PFTs were available for 15 of the 23 patients with fibrotic-like lesions, all of whom had complete data for the reported pulmonary function parameters. Mean predicted FEV_1_, FVC, FEV_1_/FVC, and DLCO values were 84.3%, 78.7%, 82.8%, and 69.3%, respectively (Table 6). The 6MWT was performed in 68 patients at 3–6 months, with a mean distance of 419.5 metres (85.5% predicted), a baseline SpO_2_ of 95.7%, and a mean minimum SpO_2_ of 92.4%. At 18–24 months, 10 patients with fibrotic-like lesions completed the 6MWT, with a mean walking distance of 438.8 metres (89.5% predicted), a baseline SpO_2_of 94.8%, and a minimum SpO_2_ of 89.5% (Table 6).

## 4. Discussion

In this ambispective, longitudinal cohort study of hospitalised patients with OP-COVID-19, we characterised the clinical, radiological, and functional evolution over two years and identified factors associated with long-term fibrotic-like sequelae. OP-COVID-19 was diagnosed in 4.9% of hospitalised COVID-19 patients, with most showing rapid resolution of the acute inflammatory OP pattern within the first months after infection. However, at 18–24 months, 14.9% of the cohort exhibited persistent lung abnormalities, and 10.4% showed fibrotic-like lesions. Notably, among patients undergoing extended imaging follow-up, no statistically detectable late radiological progression was observed.

Consistent with previous studies of both cryptogenic and OP-COVID-19, inflammatory radiological abnormalities tended to resolve early in the disease course in most patients [2,7,8,10]. In our study, fibrotic-like lesions were observed in 10.4% (23/221) of patients with an ascertainable final radiological outcome on follow-up imaging. The higher proportion of these lesions observed among those assessed with HRCT at 18–24 months (52.3%) reflects selective follow-up of patients with persistent or more slowly resolving abnormalities and should not be interpreted as representative of the overall cohort. The proportion of fibrotic-like lesions in our cohort was lower than that reported in the only study specifically addressing long-term outcomes in OP-COVID-19, in which up to 57% of patients showed fibrotic-like abnormalities at 12 months [10]. This discrepancy is likely explained by differences in the definition of fibrotic lesions [15]. While the previous study adopted a broader definition that included parenchymal bands and pleural retraction, we applied a more restrictive definition limited to traction bronchiectasis or bronchiolectasis and/or architectural distortion with volume loss. Although both cohorts were broadly comparable in demographic characteristics, the previous OP-COVID-19 cohort [10] included patients with more severe acute disease, as reflected by higher rates of ICU admission (42% vs. 31%) and use of mechanical ventilation (20% vs. 15.9%), together with a higher proportion of pre-existing respiratory disease, including bronchiectasis, which may have confounded the interpretation of traction bronchiectasis as a post-COVID sequela.

Importantly, among patients undergoing extended imaging follow-up, no statistically detectable late radiological progression was observed. The increase in bronchial dilatation and other structural abnormalities observed from the initial HRCT through follow-up likely reflects post-inflammatory evolution and cohort selection effects. Overall, the persistence of structural abnormalities at 18–24 months, together with no statistically detectable late radiological progression among patients with paired HRCT examinations, is consistent with predominantly post-inflammatory structural sequelae.

In the absence of OP-specific cohorts with longer follow-up, evidence from larger heterogeneous post-COVID studies provides the best reference available [16,17]. These studies have generally reported limited radiological change after the first year, with minimal evidence of late progression [18,19,20,21,22]. Overall, our findings are consistent with these observations, suggesting that late fibrotic progression is uncommon.

While the association between greater acute disease severity and long-term fibrotic sequelae after COVID-19 has been consistently reported in both general post-COVID cohorts [18,19,20] and OP-specific studies [10], acute disease severity alone may not fully explain the risk of long-term lung abnormalities. In our study, the need for respiratory support during the acute phase was independently associated with the presence of fibrotic-like lesions, underscoring the close relationship between initial disease severity and long-term structural sequelae in OP-COVID-19. Beyond clinical severity, bronchial dilatation and architectural distortion or volume loss on HRCT were independently associated with fibrotic-like lesions at 18–24 months in the exploratory multivariable model, suggesting that early radiological markers of structural damage may provide additional prognostic information in patients with OP-COVID-19.

Beyond these prognostic factors, persistent viral replication or delayed viral clearance has been proposed as a potential mechanism underlying post-COVID manifestations. Early antiviral treatment may therefore mitigate subsequent tissue injury by reducing viral burden and the associated inflammatory response. Previous studies have reported faster viral clearance and improved clinical outcomes with remdesivir [23,24]. Consistent with this hypothesis, a prospective study of 1966 patients followed for one year showed that remdesivir treatment during acute COVID-19 was associated with a shorter duration of persistent symptoms, with similar findings observed for dexamethasone [25]. Other antivirals, including molnupiravir and nirmatrelvir–ritonavir, have also been associated with a reduced risk of post-acute sequelae [26,27]. However, whether these treatments specifically reduce the risk of organising pneumonia or subsequent fibrotic-like pulmonary abnormalities remains uncertain.

In our cohort, corticosteroid treatment was individualised according to disease severity and clinical-radiological evolution. Recent observational evidence suggests that dexamethasone treatment during acute COVID-19 may be associated with better subsequent pulmonary outcomes, including higher DLCO and fewer fibrotic changes [28]. In contrast, in an OP-COVID-19 cohort, Cuerpo et al. found that higher cumulative corticosteroid exposure was associated with persistent fibrotic-like changes at one year, although this association may reflect confounding by indication, as patients with more severe disease were more likely to receive higher doses [10].

The epidemiological context of our cohort should also be considered. Patients were included during the early pandemic period, before widespread vaccination and the emergence of later SARS-CoV-2 variants. Subsequent pandemic waves were characterised by increasing population immunity and lower disease severity associated with later variants such as Omicron [29]. Furthermore, vaccination and pandemic-era changes have been associated with a progressive reduction in the risk of post-acute sequelae [30,31]. Therefore, the prevalence and long-term radiological outcomes of OP-COVID-19 observed in our cohort may not be directly generalisable to vaccinated populations or infections caused by later variants. Whether these changes specifically reduce the risk of persistent fibrotic-like pulmonary abnormalities remains uncertain.

The functional findings observed during follow-up support generally preserved pulmonary function in patients with OP-COVID-19. Among patients with available pulmonary function tests, spirometric parameters were largely preserved, whereas DLCO was mildly reduced in patients assessed during follow-up, including the subset of patients with fibrotic-like lesions assessed at 18–24 months. This pattern is consistent with previous OP-COVID-19 data showing that reduced DLCO is the predominant functional abnormality during follow-up, despite relatively preserved lung volumes [10,32,33,34]. This dissociation suggests that residual OP-COVID-19 sequelae may affect gas transfer more than ventilatory mechanics.

From a clinical perspective, our findings support a risk-stratified follow-up strategy in patients with OP-COVID-19. Most patients demonstrate substantial radiological resolution within the first year. After hospital discharge, only 7.4% of patients required home oxygen therapy, which was discontinued in all cases within the first month. Importantly, among patients undergoing extended imaging follow-up, no statistically detectable late radiological progression was observed, suggesting that the need for routine imaging beyond this timepoint could be individualised in clinically stable patients rather than systematically performed. However, given the observational, single-centre design and selective imaging follow-up of our study, this finding should be interpreted cautiously and does not establish a definitive imaging surveillance strategy. In contrast, patients with severe acute disease and early structural abnormalities on HRCT, such as bronchial dilatation or architectural distortion, represent a higher-risk subgroup for persistent fibrotic-like sequelae and may benefit from closer clinical and functional follow-up.

### Strengths and Limitations of the Study

This study has several strengths, including its ambispective design, long-term structured follow-up over 24 months, and focus on a well-defined and homogeneous OP-COVID-19 phenotype, rather than a heterogeneous post-COVID-19 population with diverse interstitial lung abnormalities. This phenotypic homogeneity strengthens internal validity and enables a more specific characterisation of the long-term clinical, functional, and radiological trajectory of OP-COVID-19. Standardised diagnostic criteria, uniform treatment strategies, and multidisciplinary evaluation, including assessment by expert thoracic radiologists, further enhance the robustness of the study. However, some limitations should be acknowledged. These include the single-centre design, which may limit generalisability to other populations and healthcare settings. In addition, the study was conducted during the early phase of the COVID-19 pandemic, when circulating SARS-CoV-2 variants, disease severity, and therapeutic strategies differed from those of later pandemic waves. SARS-CoV-2 vaccination status was not systematically collected, and its potential impact on long-term outcomes could therefore not be assessed. However, most patients were included before widespread vaccination coverage, as the study period (March 2020–February 2021) largely preceded the implementation of population-based vaccination campaigns. Therefore, the findings may not be fully generalisable to patients infected with subsequent variants. Although the diagnosis of OP was based on established clinical and radiological criteria, the absence of histopathological confirmation may have led to potential misclassification in a minority of cases. In addition, follow-up imaging was not systematically performed in all patients and was often discontinued after radiological normalisation, which may have introduced selection bias. The cumulative corticosteroid dose was not calculated, as corticosteroid treatment was adjusted individually according to disease severity and clinical-radiological evolution. In addition, the multivariable analysis was exploratory and based on only 23 outcome events, resulting in limited statistical precision and wide confidence intervals for some estimates. Furthermore, cumulative corticosteroid exposure could not be reliably quantified because treatment was individualised. Finally, functional assessments were not available in all patients, which may have reduced the power to fully characterise long-term functional outcomes.

## 5. Conclusions

In most patients, OP-COVID-19 is associated with favourable long-term pulmonary outcomes, with substantial radiological resolution and persistent interstitial abnormalities or fibrotic-like lesions in a minority of cases. Among patients undergoing extended imaging follow-up, no statistically detectable late radiological progression was observed. Greater initial disease severity and early structural abnormalities on HRCT were associated with long-term fibrotic-like outcomes, identifying a higher-risk subgroup. These findings support a risk-stratified follow-up strategy, with closer clinical and functional monitoring for patients with severe acute disease or early structural lung abnormalities, while routine prolonged imaging may be individualised in clinically stable patients.

## Figures and Tables

**Table 1:** Baseline characteristics of hospitalised patients with organising pneumonia secondary to COVID-19 (*n* = 271).

Variable	Measurement
Age, mean (SD)	64.7 (13.2)
Male sex, *n* (%)	183 (67.5)
Smoking status, *n* (%)	
Former or current smoker	86 (31.7)
Never smoker	185 (68.3)
Comorbidities, *n* (%)	
Obesity	61 (22.5)
Dyslipidaemia	123 (45.4)
Hypertension	146 (53.9)
Diabetes	81 (29.9)
COPD	26 (9.6)
Asthma	12 (4.8)
Bronchiectasis	5 (1.9)
Coronary artery disease	15 (5.5)
Atrial fibrillation	15 (5.5)
Chronic kidney disease	17 (6.3)
Cancer	15 (5.5)
Immunosuppression	14 (5.2)
Laboratory tests, median (IQR) ^†^	
Ferritin, ng/mL	963 (562–1589)
Fibrinogen, mg/dL	500 (399–500)
CRP, mg/dL	12 (8–18)
D-dimer at admission, ng/mL	857 (540–1322)
Peak D-dimer, ng/mL	2120 (1177–4152)
IL-6, pg/mL	47 (22–121)
NT-proBNP, pg/mL	292 (123–804)
LDH, U/L	347 (268–449)
Hospitalisation	
Length of stay (days), median (IQR)	24 (16–33)
ICU admission, *n* (%)	84 (31.0)
Respiratory support at admission, *n* (%)	125 (46.1)
Non-invasive ventilation	28 (10.3)
Continuous positive airway pressure	20 (7.4)
High-flow nasal cannula	34 (12.5)
Invasive mechanical ventilation	43 (15.9)
Discharge data	
Baseline SpO_2_, mean (SD)	96 (1.4)
Home oxygen therapy, *n* (%)	20 (7.4)
Corticosteroid treatment at hospital admission, *n* (%) ^††^	
Dexamethasone	209 (77.1)
Methylprednisolone	62 (22.9)

^†^ Data available for 256 patients; values represent peak values or values recorded at ICU admission during hospitalisation, unless otherwise specified. ^††^ All patients with OP-COVID-19 received corticosteroid treatment during hospitalisation, according to the institutional protocol (see Section 2). Abbreviations: COPD: chronic obstructive pulmonary disease, ICU: intensive care unit, CRP: C-reactive protein, NT-proBNP: N-terminal pro-brain natriuretic peptide, IL-6: interleukin-6, LDH: lactate dehydrogenase, SpO_2_: Peripheral Oxygen Saturation, IQR: interquartile range, SD: standard deviation.

**Table 2:** Follow-up high-resolution computed tomography findings in patients with organising pneumonia secondary to COVID-19.

	Initial HRCT # (*n* = 271)	3–6 Months (*n* = 228)	9–12 Months (*n* = 124)	18–24 Months (*n* = 44)
Main radiological findings				
Ground-glass opacities	269 (99.3)	218 (95.6) *	101 (81.5) *	33 (75.0) *
Consolidation	268 (98.9)	10 (4.4) *	0 (0.0) *	0 (0.0) *
Peribronchovascular opacities	229 (84.5)	12 (5.3) *	2 (1.6) *	0 (0.0) *
Parenchymal bands	211 (77.9)	150 (65.8) *	61 (49.2) *	15 (34.1) **
Reticular pattern	172 (63.5)	114 (50.0) *	73 (58.9)	32 (72.7)
Crazy-paving pattern	147 (54.2)	6 (2.6) *	1 (0.8) *	0 (0.0) *
Reversed halo sign	83 (30.6)	0 (0.0) *	0 (0.0) *	0 (0.0) *
Architectural distortion and/or volume loss	82 (30.3)	50 (21.9)	37 (29.8)	23 (52.3)
Bronchial dilatation/traction bronchiectasis ^†^	30 (11.1)	62 (27.2) *	51 (41.1) *	23 (52.3) *
Mosaic pattern	27 (10.0)	43 (18.9) *	23 (18.7)	10 (22.7)
Perilobular opacities	16 (5.9)	0 (0.0) *	0 (0.0) *	0 (0.0) *
Distribution				
Bilateral	268 (98.9)	150 (65.8)	59 (47.6)	28 (63.6)
Diffuse (central and peripheral)	238 (87.8)	14 (6.1)	2 (1.6)	0 (0.0)
All fields	207 (76.4)	13 (5.7)	2 (1.6)	1 (2.4)
Middle and lower lung fields	41 (15.1)	46 (20.2)	16 (12.9)	6 (14.3)
Lower fields	20 (7.4)	145 (63.6)	82 (66.1)	30 (68.2)
Upper and middle lung fields	3 (1.1)	6 (2.6)	5 (4.0)	2 (4.6)
Other radiological findings ^¶^				
Pulmonary embolism	20 (7.4)			
Vascular dilatations in GGO areas	28 (10.3)			
Pulmonary artery dilatation (>30 mm)	25 (9.2)			
Emphysema	19 (7.0)			
Pulmonary nodules	4 (1.5)			

Data are expressed as *n* (%). Abbreviations: HRCT, high-resolution computed tomography; GGO, ground-glass opacities. # HRCT performed at the time of organising pneumonia diagnosis; ^†^ Bronchial dilatation observed at baseline was classified as traction bronchiectasis only when confirmed on follow-up HRCT at ≥3 months, to distinguish potentially reversible bronchial dilatation from persistent fibrotic bronchial distortion; ^¶^ These findings were assessed only on initial HRCT. Paired comparisons of the main radiological findings between the initial HRCT and each follow-up examination (3–6, 9–12, and 18–24 months) were performed using McNemar’s test for dichotomous variables. Only patients with complete paired data at each time point were included. Reported *p*-values represent within-patient changes compared with initial values. In addition, exploratory paired comparisons between 9–12 and 18–24 months were performed in patients with available longitudinal imaging data. Other imaging features, including lesion distribution and additional radiological findings, were summarised descriptively but were not included in statistical comparisons. * *p* < 0.05 vs. initial ** *p* < 0.05 vs. 9–12 months.

**Table 3:** Comparison of demographic, hospitalisation, and clinical follow-up characteristics according to the presence of fibrotic-like lesions at 18–24 months in patients with organising pneumonia secondary to COVID-19 (*n* = 221) *.

Variables	No Fibrotic-like Lesions (*n* = 198)	Fibrotic-like Lesions (*n* = 23)	*p* Value
Demographics			
Age, mean (SD)	64.4 (12.5)	70.1 (11.3)	0.039
Male, *n* (%)	135 (68.2)	16 (69.6)	0.977
Never smoked, *n* (%)	135 (68.2)	15 (65.2)	0.822
Comorbidities, *n* (%)			
Obesity	45 (22.0)	7 (30.4)	0.358
Dyslipidaemia	90 (45.5)	11 (47.8)	0.857
Hypertension	112 (56.6)	12 (52.2)	0.719
Diabetes	59 (29.8)	6 (26.1)	0.679
COPD	22 (11.1)	0 (0.0)	0.143
Asthma	10 (4.9)	2 (8.7)	0.696
Bronchiectasis	3 (1.4)	0 (0.0)	1.000
Coronary artery disease	12 (5.8)	0 (0.0)	0.378
Atrial fibrillation	10 (4.9)	0 (0.0)	0.372
Chronic kidney disease	9 (4.3)	2 (8.7)	0.609
Cancer	12 (5.8)	2 (8.7)	0.640
Immunosuppression	10 (4.8)	1 (4.3)	0.907
Laboratory parameters during hospitalisation			
Ferritin **, median (IQR), ng/mL [*n* = 190/21]	959.5 (559–1563)	1174.0 (875–2475)	0.028
Fibrinogen, median (IQR), mg/dL [*n* = 83/8]	450.0 (350–500)	500.0 (384–500)	0.170
D-dimer at admission, median (IQR), ng/mL [*n* = 190/23]	810.0 (537–1254)	1200 (958–3200)	0.002
D-dimer **, median (IQR), ng/mL [*n* = 193/23]	1980.0 (1160–3725)	4150.0 (1581–5120)	0.018
CRP **, median (IQR), mg/dL [*n* = 193/23]	12.0 (9–18)	12.0 (6.5–18)	0.904
IL-6 **, median (IQR), pg/mL [*n* = 182/21]	45.1 (22–113)	42.0 (27–95)	0.899
NT-proBNP, median (IQR), pg/mL [*n* = 132/15]	269.0 (123–663)	450.0 (80–816)	0.654
LDH, median (IQR), U/L [*n* = 192/22]	344 (256–448)	369.0 (271–545)	0.278
Length of stay, median (IQR), days [*n* = 198/23]	22 (16–31)	37 (30–49)	<0.001
ICU admission, *n* (%)	59 (29.8)	17 (73.9)	<0.001
Home oxygen therapy at discharge, *n* (%)	12 (6.1)	8 (34.8)	<0.001
Respiratory support during acute phase, *n* (%)	87 (43.9)	19 (82.6)	<0.001
High-flow nasal cannula	22 (11.1)	3 (13.0)	0.725
Non-invasive ventilation	23 (11.6)	2 (8.7)	1.000
Continuous positive airway pressure	13 (6.6)	4 (17.4)	0.077
Invasive mechanical ventilation	31 (15.7)	10 (43.5)	0.001
Corticosteroid treatment, *n* = 198/23			
Total duration of corticosteroid treatment, weeks, mean (SD)	6.3 (2.4)	10.5 (2.4)	<0.001

Values are presented as mean (SD) or median (IQR), *n* (%) or as indicated. Abbreviations: COPD: chronic obstructive pulmonary disease, ICU: intensive care unit, CRP: C-reactive protein, IL-6: interleukin-6, LDH: lactate dehydrogenase, NT-proBNP: N-terminal prohormone of brain natriuretic peptide, SD: standard deviation; IQR: interquartile range. * Of the 271 patients diagnosed with organising pneumonia secondary to COVID-19, 43 were excluded from follow-up, 36 were lost to follow-up, and 7 died before follow-up. A total of 228 patients were therefore included in the longitudinal cohort. For the present analysis, fibrotic-like lesion status at the end of radiological follow-up could be determined in 221 patients (198 without and 23 with fibrotic-like lesions); 7 patients with unknown final radiological outcome were excluded. Patients without fibrotic-like lesions included both those with no residual fibrotic lesions and those whose follow-up was discontinued after HRCT normalisation. ** Values represent peak values or values recorded at ICU admission during hospitalisation.

**Table 4:** Initial high-resolution computed tomography findings according to the presence of fibrotic-like lesions at 18–24 months in patients with organising pneumonia secondary to COVID-19 (*n* = 221) *.

Variables	No Fibrotic-like Lesions (*n* = 198)	Fibrotic-like Lesions (*n* = 23)	*p* Value
GGO, *n* (%)	197 (99.5)	23 (100)	1.000
Peribronchovascular opacities, *n* (%)	162 (81.8)	22 (95.7)	0.137
Reticular pattern, *n* (%)	119 (60.1)	20 (87.0)	0.012
Crazy-paving pattern, *n* (%)	101 (51.0)	18 (78.3)	0.013
Consolidation, *n* (%)	195 (98.5)	23 (100)	1.000
Reversed halo sign, *n* (%)	60 (30.3)	9 (39.1)	0.387
Parenchymal bands, *n* (%)	154 (77.8)	22 (95.7)	0.053
Bronchial dilatation, *n* (%)	15 (7.6)	7 (30.4)	0.003
Architectural distortion and/or volume loss, *n* (%)	41 (20.7)	14 (60.9)	<0.001
Diffuse distribution (central and peripheral), *n* (%)	170 (85.9)	22 (95.7)	0.221
Bilateral distribution, *n* (%)	195 (98.5)	23 (100)	1.000
Distribution by lung fields, *n* (%)			
Upper and middle lung fields	1 (0.5)	0 (0)	NA
Middle and lower lung fields	30 (15.2)	0 (0)	NA
Lower fields	19 (9.6)	0 (0)	NA
All fields	148 (74.7)	23 (100)	0.003
Pulmonary embolism, *n* (%)	17 (8.6)	2 (8.7)	1.000
Vascular dilatations in GGO areas, *n* (%)	21 (10.6)	3 (13.0)	0.731
Mosaic pattern, *n* (%)	22 (11.1)	2 (8.7)	1.000
Emphysema, *n* (%)	14 (7.1)	3 (13.0)	0.387
Pulmonary nodules, *n* (%)	3 (1.5)	1 (4.3)	0.358
Pulmonary artery diameter > 30 mm, *n* (%)	16 (8.1)	5 (21.7)	0.051

Values are presented as *n* (%). Abbreviations: NA, not applicable; GGO: Ground-glass opacities. * Of the 271 patients diagnosed with organising pneumonia secondary to COVID-19, 43 were excluded from follow-up, 36 were lost to follow-up, and 7 died before follow-up. A total of 228 patients were therefore included in the longitudinal cohort. For the present analysis, fibrotic-like lesion status at the end of radiological follow-up could be determined in 221 patients (198 without and 23 with fibrotic-like lesions); 7 patients with unknown final radiological outcome were excluded. Patients without fibrotic-like lesions included both those with no residual fibrotic lesions and those whose follow-up was discontinued after HRCT normalisation.

**Table 5:** Exploratory multivariable logistic regression analysis of factors associated with fibrotic-like lesions at 18–24 months in patients with organising pneumonia secondary to COVID-19.

Variable	OR	95% CI	*p*-Value
Respiratory support during acute phase *	5.79	1.81–18.52	0.003
Bronchial dilatation on initial HRCT	3.50	1.10–11.11	0.033
Architectural distortion and/or volume loss on initial HRCT	4.72	1.80–12.35	0.002

* Respiratory support included high-flow nasal cannula, non-invasive ventilation, continuous positive airway pressure, and invasive mechanical ventilation. HRCT: High-resolution computed tomography; CI: confidence interval; OR: odds ratio. The model was built using patients with fibrotic-like lesions at 18–24 months (*n* = 23) as the outcome group and those without fibrotic-like lesions (*n* = 198) as the reference group. The latter included patients with no residual fibrotic lesions followed to 18–24 months and those whose follow-up was discontinued after HRCT normalisation. Other clinical, biochemical, and radiological variables (including age, length of hospital stay, corticosteroid treatment duration, ferritin, IL-6, D-dimer, reticular pattern, crazy-paving pattern, parenchymal bands, and involvement of all lung fields) were evaluated but showed no independent association and were not retained in the final model.

**Table 6:** Pulmonary function and six-minute walk test during the follow-up of patients with organising pneumonia secondary to COVID-19.

Variable	3–6 Months	18–24 Months
Pulmonary function tests *	*n* = 88	*n* = 15
FEV_1_, % predicted	85.0 (17.0)	84.3 (15.2)
FVC, % predicted	86.3 (15.9)	78.7 (14.5)
FEV_1_/FVC, %	77.4 (7.6)	82.8 (6.4)
DLCO, % predicted	76.0 (19.6)	69.3 (22.3)
KCO, % predicted	89.0 (41.0)	81.2 (18.1)
**Six-minute walk test**	*n* = 68	*n* = 10
Distance, m	419.5 (87.5)	438.8 (59.8)
Distance, % predicted	85.5 (18.9)	89.5 (9.5)
Baseline SpO_2_, %	95.7 (1.9)	94.8 (1.5)
Minimum SpO_2_, %	92.4 (3.1)	89.5 (2.7)

Values are presented as mean (SD). Abbreviations: FEV_1_: forced expiratory volume in one second, FVC: forced vital capacity, DLCO: diffusion capacity for carbon monoxide, KCO: transfer coefficient for carbon monoxide, SpO_2_: peripheral capillary oxygen saturation. * At each time point, all patients included in the pulmonary function test analysis had complete data for both spirometry and diffusing capacity measurements.

## Data Availability

The data presented in this study are available from the corresponding author upon reasonable request.

## Supplementary Materials

The following supporting information can be downloaded at: https://www.mdpi.com/article/10.3390/jcm15186999/s1, Table S1. Exploratory paired longitudinal analysis of radiological findings.
