# Supplementary material for: Clinical and Radiological Outcomes of Organising Pneumonia Secondary to COVID-19: A 2-Year Longitudinal Cohort Study
Source: J Clin Med. 2026 Sep 10;15(18):6999. doi: 10.3390/jcm15186999 (PMC13607309; doi:10.3390/jcm15186999)
Supplement: Supplementary file 1 [file jcm-15-06999-s001.zip › jcm-4478292-supplementary.pdf]

Supplementary Table S1. Exploratory paired longitudinal analysis of radiological findings.

A. Initial HRCT vs. 3 - 6 months (n=228)

Radiological finding	Paired n	Initial HRCT n (%)	3–6 months n (%)	p value
Ground-glass opacities	228	227 (99.6)	218 (95.6)	0.012
Consolidation	228	225 (98.7)	10 (4.4)	<0.001
Peribronchovascular opacities	228	191 (83.8)	12 (5.3)	<0.001
Parenchymal bands	228	182 (79.8)	150 (65.8)	0.001
Reticular pattern	228	143 (62.7)	114 (50.0)	0.001
Crazy-paving	228	122 (53.5)	6 (2.6)	<0.001
Reversed halo sign	228	70 (30.7)	0 (0.0)	<0.001*
Architectural distortion/volume loss	228	58 (25.4)	50 (21.9)	0.350
Bronchial dilatation/traction bronchiectasis	228	26 (11.4)	62 (27.2)	<0.001
Mosaic pattern	228	25 (11.0)	43 (18.9)	0.011
Perilobular opacities	228	16 (7.0)	0 (0.0)	<0.001*

B. Initial HRCT vs. 9 - 12 months (n=124)

Radiological finding	Paired n	Initial HRCT n (%)	9–12 months n (%)	p value
Ground-glass opacities	124	123 (99.2)	101 (81.5)	<0.001
Consolidation	124	122 (98.4)	0 (0.0)	<0.001*
Peribronchovascular opacities	124	105 (84.7)	2 (1.6)	<0.001
Parenchymal bands	124	97 (78.2)	61 (49.2)	<0.001
Reticular pattern	124	83 (66.9)	73 (58.9)	0.154
Crazy-paving	124	74 (59.7)	1 (0.8)	<0.001
Reversed halo sign	124	36 (29.0)	0 (0.0)	<0.001*
Architectural distortion/volume loss	124	34 (27.4)	37 (29.8)	0.736
Bronchial dilatation/traction bronchiectasis	124	17 (13.7)	51 (41.1)	<0.001
Mosaic pattern	124	16 (12.9)	23 (18.7)	0.210
Perilobular opacities	124	10 (8.2)	0 (0.0)	0.002*

C. Initial HRCT vs. 18 - 24 months (n=44)

Radiological finding	Paired n	Initial HRCT n (%)	18–24 months n (%)	p value
Ground-glass opacities	44	43 (97.7)	33 (75.0)	0.006
Consolidation	44	44 (100.0)	0 (0.0)	<0.001*
Peribronchovascular opacities	44	36 (81.8)	0 (0.0)	<0.001*
Parenchymal bands	44	37 (84.1)	15 (34.1)	<0.001
Reticular pattern	44	31 (70.5)	32 (72.7)	1.000
Crazy-paving	44	29 (65.9)	0 (0.0)	<0.001*
Reversed halo sign	44	13 (29.5)	0 (0.0)	<0.001*
Architectural distortion/volume loss	44	16 (36.4)	23 (52.3)	0.065
Bronchial dilatation/traction bronchiectasis	44	11 (25.0)	23 (52.3)	0.002
Mosaic pattern	44	6 (13.6)	10 (22.7)	0.344
Perilobular opacities	44	3 (6.8)	0 (0.0)	0.250*

D. 9 - 12 months vs. 18 - 24 months (n=44)

Radiological finding	Paired n	9 – 12 months n (%)	18–24 months n (%)	p value
Ground-glass opacities	44	40 (90.9)	33 (75.0)	0.065
Consolidation	44	0 (0)	0 (0)	NA
Peribronchovascular opacities	44	1 (2.3)	0 (0)	1.000*
Parenchymal bands	44	28 (63.6)	15 (34.1)	0.002
Reticular pattern	44	31 (70.5)	32 (72.7)	1.000
Crazy-paving	44	1 (2.3)	0 (0)	1.000*
Reversed halo sign	44	0 (0)	0 (0)	NA
Architectural distortion/volume loss	44	24 (54.5)	23 (52.3)	1.000
Bronchial dilatation/traction bronchiectasis	44	28 (63.6)	23 (52.3)	0.063
Mosaic pattern	44	9 (20.5)	10 (22.7)	1.000
Perilobular opacities	44	0 (0)	0 (0)	NA

Values are presented as n (%). Analyses included only patients with paired HRCT observations, with baseline frequencies referring to the same paired subset. Comparisons were considered exploratory because no adjustment for multiple comparisons was applied.

*Exact two-sided p-values for variables with no positive cases at follow-up were calculated from the binomial distribution of discordant pairs.
